# How can scientists bring research to use: the HENVINET experience

**DOI:** 10.1186/1476-069X-11-S1-S2

**Published:** 2012-06-28

**Authors:** Alena Bartonova

**Affiliations:** 1 NILU - Norwegian Institute for Air Research, POB 100, 2027 Kjeller, Norway

## Abstract

**Background:**

Health concerns have driven the European environmental policies of the last 25 years, with issues becoming more complex. Addressing these concerns requires an approach that is both interdisciplinary and engages scientists with society. In response to this requirement, the FP6 coordination action “Health and Environment Network” HENVINET was set up to create a permanent inter-disciplinary network of professionals in the field of health and environment tasked to bridge the communication gap between science and society. In this paper we describe how HENVINET delivered on this task.

**Methods:**

The HENVINET project approached the issue of inter-disciplinary collaboration in four ways. (1) The Drivers-Pressures-State-Exposure-Effect-Action framework was used to structure information gathering, collaboration and communication between scientists in the field of health and the environment. (2) Interactive web-based tools were developed to enhance methods for knowledge evaluation, and use these methods to formulate policy advice. (3) Quantification methods were adapted to measure scientific agreement. And (4) Open architecture web technology was used to develop an information repository and a web portal to facilitate collaboration and communication among scientists.

**Results:**

Twenty-five organizations from Europe and five from outside Europe participated in the Health and Environment Network HENVINET, which lasted for 3.5 years. The consortium included partners in environmental research, public health and veterinary medicine; included medical practitioners and representatives of local administrations; and had access to national policy making and EEA and WHO expertise. Dedicated web-based tools for visualisation of environmental health issues and knowledge evaluation allowed remote expert elicitation, and were used as a basis for developing policy advice in five health areas (asthma and allergies; cancer; neurodevelopmental disorders; endocrine disruption; and engineered nanoparticles in the environment). An open searchable database of decision support tools was established and populated. A web based social networking tool was developed to enhance collaboration and communication between scientists and society.

**Conclusions:**

HENVINET addressed key issues that arise in inter-disciplinary research on health and environment and in communicating research results to policy makers and society. HENVINET went beyond traditional scientific tools and methods to bridge the communication gap between science and policy makers. The project identified the need for a common framework and delivered it. It developed and implemented a variety of novel methods and tools and, using several representative examples, demonstrated the process of producing politically relevant scientific advice based on an open participation of experts. It highlighted the need for, and benefits of, a liaison between health and environment professionals and professionals in the social sciences and liberal arts. By adopting critical complexity thinking, HENVINET extended the traditional approach to environment and health research, and set the standard for current approaches to bridge the gap between science and society.

## Background

Human health linked with environmental quality has been on the European agenda for many years, leading to significant improvement in many areas. In 2003, the SCALE process lead to the EU Environment and Health Strategy, developing a long-term vision seeking to address the links between poor health and environmental problems, and to "reduce diseases linked to environmental factors". The following Environment Health Action Plan 2004-2010 (EHAP) [1] brought together current knowledge and identified 13 priority areas, of which four were dedicated to reviewing policies and improving collaboration and communication. The EHAP acknowledges multi-causality in environment and health, identifies priority health endpoints, and calls for a high level of inter-disciplinary knowledge and an ability to communicate within and between science and decision making community. The EU 6^th^ Framework program responded by a call for proposals to “create a permanent network of professionals in environment and health”, specifically asking to address the EHAP health priorities (asthma and allergies, childhood cancer, neurodevelopmental disorders, and endocrine disruption). The call was answered by a 3.5 year HENVINET project.

The project established a wide collaboration between many disciplines and sectors, and it can serve as a comprehensive example of “inter-disciplinary” collaboration [2]. Inter-disciplinarity requires firm commitment from the participants: the complexities of each discipline have to be understood and respected by all. The target – prevention of environmentally related diseases –requires strong policy support, as only those issues recognized by policy makers are addressed. The health and environment community aims to support current policy making, and to point out new threats. In each of the medical and the environmental professions, sectoral mechanisms for policy support are in place. The health and environment field includes both these communities, and has implications to other sectors as well. Creating support mechanisms is thus more difficult.

This paper is an introduction to the in-depth reports on HENVINET in this Supplement. It provides an overview of our activities, and describes our experiences. It reflects on how our approach to inter-disciplinarity led to a shift in focus from traditional research instruments and methods to approaches that better address collaboration and communication.

### The HENVINET project

The consortium comprised 25 European partners and five partners outside Europe, with experts. from the risk assessment community, environmental and air pollution epidemiology, clinical practice and public health, and from environmental institutes dealing mainly with air pollution (for partner list, see http://www.henvinet.eu). Through an advisory group, HENVINET also involved decision-makers from local and national administrations, and international organizations. The total range of expertise was somewhat broader than in previous activities, such as the AIRNET [3], [4] or the PINCHE [5] networks. The consortium incorporated a social sciences expertise from the 2^nd^ year of the project.

**Figure 1:**
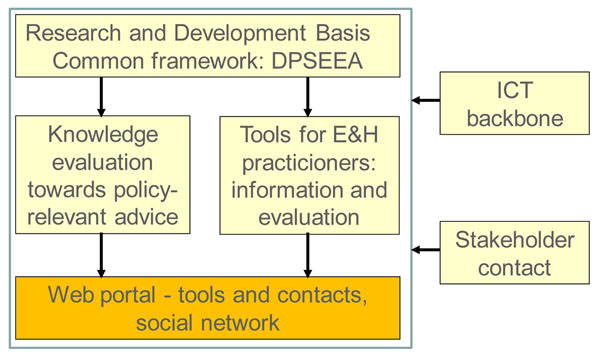
**The HENVINET elements.** DPSEEA – drivers, pressures, state, exposure, effect, action. ICT – information and communication technologies. E&H – environment and health.

The project was done through several integrated strands of work (Figure 1), all using a common framework. The research-oriented “Knowledge Evaluation” and “Tools for Practitioners” were supported by a technological backbone and a dissemination and communication activity (“Stakeholder Contact”). The Knowledge Evaluation was organized in four topic groups, each addressing one of the EHAP health priorities.

## Approach and challenges

In order to “create a permanent network”, we had to solve several methodological issues: (1) develop a tool for collaboration and communication of ideas within the consortium and to the outside, (2) provide a method that would allow assessment of what science “knows”, (3) provide a way to make practical tools available, and (4) decide on how this “network” should operate. We have developed and applied tools that solve all these issues, and in this way, have created a toolbox that can be applied to different aspects of collaboration and communication between science, policy and other stakeholders.

### Tools for collaboration and communication: common framework and complexity

The fundamental element of the project was the development of a common framework. The consortium members brought to the project different experiences and traditions, often from single discipline. Such common approach is required to join these expertises for a common purpose to provide policy advice, and to maintaining coherence across the project. In the environmental disciplines, operational frameworks such as the Pressure-State-Impact or the Drivers-Pressures-State-Impact-Response (DPSIR) framework [6] have been used since the 1980s. DPSIR provides an intuitive operationalisation for a large variety of issues. Approaches of integrated environmental assessment [7] expand this concept. A clearly useful framework is the extension by WHO, the Drivers-Pressures-State-Exposure-Effect-Action (DPSEEA [8]). It puts emphasis on exposure and effect; essential factors when dealing with health [9].

Henvinet adopted the DPSEEA framework, informed by developments in integrated environmental health impact assessment[7], and used it also as a communication tool in all the topical case studies [10-16]. As Fucic et al. [16] note, the diagrams constructed along the DPSEEA to help the experts in the evaluations, provide an excellent visual communication tool, also suitable for discussions with non-experts. The framework has also been used to build a set of descriptors for a database of decision support tools [17]. Theoretical aspects that arise in application of this kind of framework, requiring collaboration of many disciplines to arrive at a common product, are addressed from the critical complexity perspective by Keune [18].

### What does science know: the way from review to policy brief

Translating research results for policy requires an understanding of the needs of each stakeholder group. Traditional research outputs such as reviews are obviously not suited for the needs of the public, or the policymakers. The process of translating research results for policy, or for the public, has been studied, but is seldom successfully carried out. The high degree of inter-disciplinarity required in the health and environment makes it difficult even for the research actors to understand each other.

A common framework is a necessary but not a sufficient requirement for such collaboration. There is also a need to reach a broad scientific consensus, and to reach an understanding of areas where the consensus cannot be attained with present knowledge. Further, the consensus or the lack of it needs to lead to appropriate actions. These needs are addressed by an expert elicitation methodology described by Keune et al [19], applied in the case studies [10-16].

### What does science know: assessing knowledge and measuring consensus

One of the starting points of the project was a search for a methodology for knowledge assessment. The consortium considered to develop a series of reviews as tools for knowledge evaluation. We soon realized that this would not provide the wide consensus needed, but only one more additional piece of evidence. We decided instead to provide only an initial knowledge status assessment (a review), and to ask experts outside the consortium for their views [19].

A specific methodological element was missing: how to evaluate consensus on the state of knowledge. The starting set of criteria for knowledge evaluation was based on nine theoretical properties of information and knowledge (such as robustness or fitness for purpose), each with a 1-5 scale of evaluation, with a description of requirements for each score. Such a complex methodology turned out difficult to apply, and led to a fragmented assessment, nearly impossible to summarize. A simpler concept was adopted [19], using a scale similar to the one used by the International Panel on Climate Change for assessment of uncertainty. In most cases, this concept is implemented through a set of questions “What is your level of confidence in the scientist’s ability to...”, with answers on a scale 1 (very low) to 5 (very high), each number on the scale described as a probability value.

To interpret the results, we need to define what constitutes consensus, or the lack of it. A methodology was adopted from [20]. They propose a mathematical measure, developed to yield a logical determination of dispersion around a category value. A Likert 5-category scale was constructed (Very High confidence (VH), High Confidence (H), Medium confidence (M), Low confidence (L), and Very Low confidence (VL)), assigning these categories ordinal values (scores): VH=5, H=4, M=3, L=2, VL=1. This allows the calculation of a *“*Consensus value” for each question. A complete lack of consensus generates a value of 0, and a complete consensus of opinion yields a value of 1. The consensus value is then interpreted together with the mean score for each question (for formulas, see [20] or [12]).

In the case studies, this method was applied to every “What is your level of confidence ...” question asked. The numbers of questions in case studies varied between 27 (Chlorpyrifos, [11]) and 63 (HexaBromoCycloDodecane, [13]). To identify areas that merit interest, we ranked the consensus values and then further explored the questions that ranked lowest, or highest.

**Figure 2:**
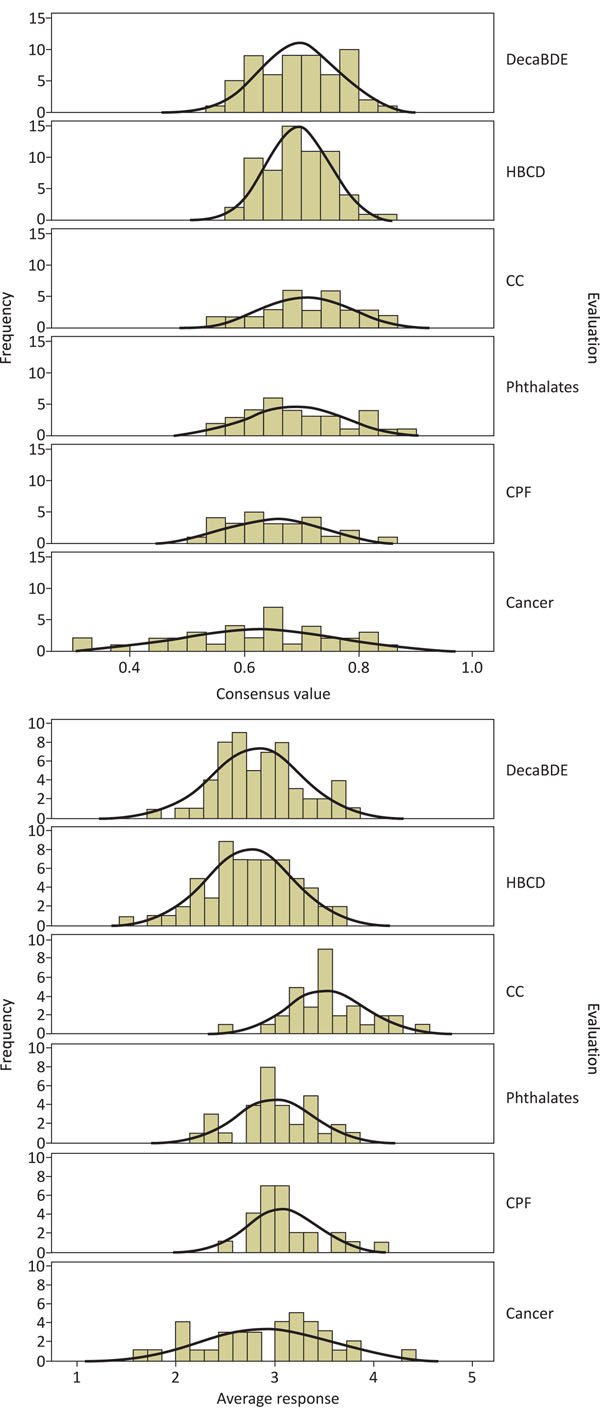
**Consensus and agreement values across the evaluations.** Frequency distribution of (1) consensus values (upper panel, values range from 0=disagreement to 1=consensus) and (2) average responses (lower panel, categories from very low=1 to very high = 5), for five HENVINET case studies – online questionnaire evaluations of (from top) DecaBromoDiphenylEther DecaBDE and HexaBromoCycloDodecane HBCD [13], Climate change CC [10], Phthalates [12], Chlorpyrifos CPF [11] and Cancer [14]. The curves represent a theoretical normal distribution, and serve as a visual aide. Number of questions in the 5 online questionnaires ranged from 27 (CPF) to 63 (HBCD).

We can compare the consensus values and scores across the different case studies (Figure 2). It appears that lowest average ranking (i.e., least average confidence that science has the knowledge) is for Brominated Flame Retardants, but on most questions, there is a relatively high level of agreement. In Climate Change [10], there is on average high confidence in available knowledge (high score), but a comparatively large spread in consensus. On Cancer, there is a large spread of confidences (scores), and the largest spread of consensus values. No data have been found in the literature that would allow us to compare these findings, and to interpret them in relation to other studies, but the results do reflect our intuitive understanding: the Brominated Flame Retardants were evaluated in a framework very similar to risk assessment, familiar to the participating experts. For Cancer, such an evaluation and the use of the DPSEEA framework have never been reported before: the result may thus reflect both the large differences in knowledge in the different elements of the framework, and the uncertainty from the relative novelty of the approach. Based on the results, we feel that this quantitative procedure provides a good basis for expert discussions leading in the next step to identification of possible actions.

### Practical tools to use research in decisions

Decision support tools (DSTs) are a special kind of research-based instruments that support the translation of research results to decision-making. We have developed a DST database [17], with descriptors derived from the DPSEEA framework.

In order to help potential users to decide whether or not a given tool is useful for their purpose, we have suggested a scheme to evaluate the tool’s applicability and ease of use. The evaluation results are included in the database. Often, users ask how a DST was validated. A formal validation lies with the DST’s author or provider, but the user needs to be informed whether or not the tool was validated, and should be able to find the validation results.

The process to create the database took almost two years. A number of issues were thoroughly discussed, such as how to translate the DPSEEA framework into descriptors that would be both general and specific enough, how to define categories of information to be included, and what descriptors are essential. Feasibility of information gathering, access rights to the database and technical implementation were other important considerations. The database is in operation, open to public and can be further built upon.

### Means to communicate: social networks

At about the midpoint of the project, we asked the project advisory board to review our activities up to that date. We received serious criticisms along the lines “more of the same”: the reviewers could see the scientific value, but did not recognize any activities that would promote the networking and communication aspects, and thus were in doubt whether the project would reach its goal. The consortium responded by brainstorming and arrived at the idea of creating a social networking tool. We have identified the essential functionalities and content, and built and promoted the tool. The process and its results are described in [21] and [22]. The tool provides also access to all the products from the project.

### Addressing future issues

It takes time to formulate scientific information in a form ready to be used for policy advice. We have provided an initial scientific assessment on selected issues that arose as knowledge gaps during the work on knowledge evaluation, but did not pursue the full HENVINET chain leading to a policy brief. In one case, we have organized a workshop to explore how a group of major stakeholders, city administration representatives, perceive scientific advice in health and environment.

Environmental risk factors for xenoestrogens and estrogen-related cancers were reviewed by Fucic et al [16]. Leijs et al. [23] reviewed thyroid hormone metabolism and the impact of environmental chemicals, and generated new data. Letasiova et al. [24] reviewed knowledge on bladder cancer, and Volkovova et al. [25] reviewed available studies on cutaneous melanoma. Cumulative risks of mixtures of chemicals were reviewed from the point of view of policy making by Sarigiannis and Hansen [26].

Keune et al [27] report on the workshop that addressed the future concerns in environmental health in urban areas. They asked what may be the consequences of climate change, and how best to address them. Cities are often charged with implementation of various legislative instruments and mitigating measures, but do not always have enough information upon which to act. The workshop provided an example where a shared future vision to maintain or improve on the quality of environmental health by 2030 allows issue framing and identifying of knowledge gaps, as well as defining actions to take.

## Context of inter-disciplinarity

HENVINET allowed the participants to develop an understanding of work in an inter-disciplinary consortium. This statement, however obvious it may seem, hides many difficulties encountered along the way. Despite the ubiquity of “inter-disciplinarity”, not many definitions are available. Aboelela et al [28] have reviewed existing literature, interviewed experts and tested a draft definition, and finally suggest the following: “*Inter-disciplinary research is any study or group of studies undertaken by scholars from two or more distinct scientific disciplines. The research is based upon a conceptual model that links or integrates theoretical frameworks from those disciplines*, *uses study design and methodology that is not limited to any one field*, *and requires the use of perspectives and skills of the involved disciplines throughout multiple phases of the research process*”. To begin with, we approached “inter-disciplinarity” intuitively, using a parallel disciplinary approach and providing an arena for information exchange (the semi-annual consortium meetings). With time, we have moved to inter-disciplinary research, and possibly beyond, towards engagement with society.

Inter-disciplinarity promotes perspectives that enable us to arrive at solutions to problems arising in “real life”, often significantly supplementing mono-disciplinary approaches. Yet this desirable state is difficult to achieve. Insights are offered by Hall et al [29] who give a systematic attention to inter-disciplinarity in health research in Canada. They note that institutions (and educational systems) are usually not set up for inter-disciplinarity. They summarize the main challenges, and suggest potential measures to promote inter-disciplinarity in health research, which seem to have general appeal. These measures fall into four categories: (1) provision of resources, (2) recognition and reward, (3) training, and (4) professional organizations. Looking at these categories one by one, we can state that (1) HENVINET obtained funding to do inter-disciplinary research. The issue (2) of recognition and reward in academic terms was a challenge for the team: to publish an inter-disciplinary review is difficult; mono-disciplinary journals may not recognize such texts as deep enough or in scope to be accepted, and when contrasted with mono-disciplinary excellence, “inter-disciplinarity” can be perceived as shallow, possibly also because not much room is available for the mono-disciplinary deliberations. Regarding (3) training, research scientists do not usually receive training in inter-disciplinarity; however, a risk assessment perspective is a good starting point. Learning about concepts in integrated assessment, or integrated environmental health impact assessment, also provides for excellent training. Addressing (4), to create a platform for a professional community was a key aim for HENVINET. Thus, moving from the intuitive, HENVINET has been implementing measures to address the four challenges. Often, this was difficult, but unlike Laberge et al. who found that the majority of scientists participating in their study [30] were sceptical to the added value of inter-disciplinarity, the HENVINET consortium has grown more and more enthusiastic with time, confirming perhaps the view of Whitfield and Reid [31] that an inter-disciplinary approach brings more insight to environmental health problems.

Moving beyond inter-disciplinarity, i.e. engaging with society and societal issues has been a goal of HENVINET. Guimarães and Funtowicz [32] give an overview of the different terms related to “trans-disciplinarity” and provide a comprehensive example of a process in governance of groundwater resources. They describe the example as follows: “… GOUVERNe process was strongly based on trans-disciplinary principles, combining hybrid methodologies, integrating social research methods with evaluation tools”. The HENVINET effort stemmed from natural and medical sciences and only at a late stage incorporated a professional in social sciences. Our approach to the trans-disciplinarity challenge was not systematic to begin with, but has moved in a similar direction to that described by these authors. The process was helped greatly by a critical complexity perspective [18] that allowed us to look at inter-disciplinarity from another angle, and provided further incentives for inter-disciplinary engagement. Another perspective, a framework that allows placing health sciences in the “Knowledge Universe”, is offered by Choi and Pak [33] who promote the appreciation of links between disciplines, the “vastness of the knowledge universe”, and identification of issues suitable to foster “multiple disciplinary efforts”.

A pertinent aspect of inter-disciplinarity in environmental health is the integration of social sciences for solving environmental health issues. In HENVINET, we have experienced a different level of understanding and ability to approach problems with the arrival of a social scientist in our midst. As Lewis [34] points out, the social scientist’s perspective provides insights that are essential to the interfacing of the scientific results with policies. Yet, Albert et al [35] report that many biomedical scientists have a negative attitude towards social sciences, claiming that the research methods do not generate valid experimental results. In HENVINET, we believe that we have generated experimental results (in the on—line evaluations), and they were essential to arrive at a valid product – the policy briefs generated in the case studies [10-15].

## Networking and the science-policy link

In HENVINET, we found it difficult to establish an inter-disciplinary network and a link between science and policy. One could argue that this was due to the lack of social sciences involvement early in the project: their perspective on processes of knowledge development and problem solving would undoubtedly have enabled us to adopt systematic approaches earlier. Several authors have offered a wider perspective, resonating well with our experiences.

Choi et al [36] looked into how to promote collaboration between scientists and policy makers. They point out that goals of scientists (in-depth disciplinary understanding) and of policy makers (to obtain public support) are divergent, that each community has their own distinct language, and that their time perspectives for finding solutions are different (policy makers work in the “now” while scientists need time to test their findings). Barriers that they identify bear similarities to those reported in the results of the HENVINET survey [22]. Some of the solutions that Choi et al [36] suggest – the role of “facilitators” to use of research in policy making – were identified also in HENVINET through a more organic development process, and were implemented in the form of a the social network portal. Promoting contact through social media network will not supersede own personal physical network. Social media may however provide access to experts and to timely and relevant information about research that confirms current policy, or point out areas of possible community pressure or client demand for research.

Traceability of information is an issue that has been mentioned as a requirement for accepting results for policy or decision making [12]. Our somewhat limited experience is that traceability of information, or the information pedigree, is one important factor in the acceptance of a “policy brief” created by scientists. This is also discussed by Eden [37], who gives an example of work of the Forest Stewardship Council’s network for environmental governance. This complex network seeks to establish a standard for forest management, acceptable to environmental, social and economic member organizations, and as in any standardization or certification process, also here the “chain of custody” or information pedigree is central. HENVINET can be seen as an attempt to establish a complex network that can employ the knowledge evaluation process to arrive at standardized policy-relevant information – the policy briefs. Being able to access information about every step of the process that led to the brief will increase the acceptance of such aggregated knowledge presentations.

For research results to be accepted by non-researchers, traceability is one consideration, but the situation is more complex. Owens [38] has been studying science-policy link for many years, using examples of different environmentally related decision making processes in the UK. In a 2005 commentary, she reflects on the potential of “research to make a difference”, and to exert influence on public policy and practice. She notes that there is a tendency to attribute the problem of “never using knowledge for the benefit of policy” to shortcomings in communication, and analyzes this premise, concluding that the linear relationship, the “technical rationality” model in which results from research/science become raw material for the policy, is inadequate. She discusses the “strategic knowledge“ model and its aspects including the choice of knowledge and the delay between results generation and their use in the policy process (when the results are used, the science may have moved forward). Owens argues that a way forward may involve a move from strategic knowledge to cognitive perspectives, acknowledging that questions may be trans-scientific and unstructured, and require different kinds of knowledge to be considered. Finally, Owens suggests employing intermediaries to seek and interpret the results of relevant academic research, since “hero researchers” who manage both an academic career and active dissemination of their research to policy communities are an unrealistic concept. She also argues for more research into the “boundary” between science and policy, and analyses the process deeper in [39] and [40]. It is easy to relate these concepts to our own experience: not least, most consortium members are familiar with some examples of a failure of the linear model, and many have served on committees that were created to take upon themselves the role of “hero researcher” moving towards the “strategic knowledge” model. The HENVINET expert elicitation process is an attempt to deal in practice with some of the potential shortcomings of the “strategic knowledge” model. To achieve fairness in terms of representativeness of scientific opinions, we needed to define criteria for eligibility of experts to be invited to the evaluation: these criteria are also a part of the final result, and an aspect of the information traceability.

Overall, the HENVINET as it has developed, has tried to become the intermediary between research results and their use for decision and policy making: some members of the consortium are the “hero scientists”, but the development of the knowledge evaluation process has changed us all towards being more perceptive to the difficulties of the “mediation” or “facilitation” process.

## The role of social media

Using social media to facilitate collaboration between science and policy is a novel approach: we were not able to find prior examples that could guide our own work. Facebook-like solutions are used only sporadically by the scientific community, and then mostly by professional societies, with much lower need for “inter-disciplinarity” of the participants, and usually with up to a few hundred of members.

A number of information and communication solutions promote scientific collaboration, as reviewed by Schleyer et al [41]. It seems quite likely that social networking will be more and more common, but to be used, it needs to bring to the participants a clear added value. The HENVINET portal provides access to tools for communication between science and policy, as far as they were developed in the project. The potential added value to the users, beyond the tools, seems unclear. As Choi et al [36] point out, the agendas of different actors vary, and there are numerous other barriers. How can these barriers be overcome for the common goal of improving environmental health? In the absence of pressing problems or agendas, there is limited interest (as an example, we have seen on an example of brominated flame retardants, that when an issue was taken on a political agenda, the portal activities have increased). We have identified and implemented core functionalities, but the resources necessary to gain a critical mass of users, and to maintain the content, are beyond our current reach. The chances that electronic social networks will supersede traditional personal networks for professional purposes seem at the moment not overwhelming. But web-based social media offer a way to carry out a dialogue also between scientific communities and between science and the society, and are thus likely to diminish the communication barriers.

## Conclusions

HENVINET addressed key issues that arise in inter-disciplinary research on health and environment and in communicating research results to policy makers. It did so by accepting that to communicate between science and society we need to go beyond traditional scientific tools and methods. The resulting dialogue between participants from complementary scientific disciplines has increased their knowledge of and respect for multiple perspectives and complexity; it also enhanced appreciation of the significance of these complexities for decision making.

The HENVINET project provides insights that may lead to lowering the existing barriers. Based in the common framework, HENVINET has developed a variety of methods and tools for collaboration and communication, and implemented them as concrete novel products. Through the web portal http://www.henvinet.eu, serving both as the repository of the methods and as a social networking tool, we hope to extend this awareness to scientists and decision makers outside the consortium.

The project has demonstrated the process of producing scientifically based politically relevant advice based on an open participation of experts. Our experience provides concrete examples of difficulties inherent in such process, but also of the added value that this process provides. To carry this work further, scientists in health and environment need to liaise with disciplines in “soft” sciences including social sciences and liberal arts. There is much more to be done in this field; we hope that our experiences will be useful to our successors.

## Competing interests

There are no competing interests.
